# Editorial: Function and Dysfunction of Complement Factor H

**DOI:** 10.3389/fimmu.2021.831044

**Published:** 2022-01-13

**Authors:** Mihály Józsi, Paul Nigel Barlow, Seppo Meri

**Affiliations:** ^1^ MTA-ELTE Complement Research Group, Eötvös Loránd Research Network (ELKH), and Department of Immunology, ELTE Eötvös Loránd University, Budapest, Hungary; ^2^ Schools of Biological Sciences and Chemistry, University of Edinburgh, Edinburgh, United Kingdom; ^3^ Department of Bacteriology and Immunology, University of Helsinki, Helsinki, Finland

**Keywords:** complement, factor H (FH), factor H-related protein (FHR), alternative pathway (AP), innate immunity

The complement system responds very quickly to danger due to the eternal vigilance of its alternative pathway (AP). In the AP, an always-on positive-feedback C3b-amplification loop (**Figure 1**) is maintained at “tick-over” level on autologous surfaces by protective membrane-bound and soluble regulators. Conversely, the unprotected surfaces of invading microbes rapidly become opsonised by AP-generated C3b, tagging them for clearance, triggering the cytolytic terminal pathway and releasing anaphylatoxins C3a and C5a. Pathogenic microbes, however, often resist attack by complement, thus avoiding elimination.

**Figure 1:**
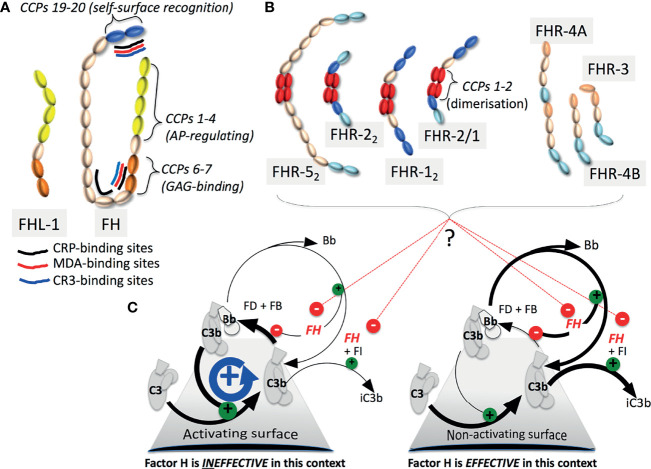
*The FH family and C3b amplification*: **(A)** FHL-1 and FH contain seven and 20 CCPs (SCRs) respectively. Functional sites on FH are indicated. **(B)** The CCPs of the FHRs are colour-coded to highlight similarity with CCPs in FH. Dimerisation-mediating CCPs 1-2, are unique to FHRs-1, 2 and 5. Of potential heterodimers, only FHR-2/1 is detectable. **(C)** In a positive-feedback cycle factor B (FB) binds nascent C3b (that can covalently attach to virtually any surface) whereupon factor D (FD) cleaves FB to yield C3b.Bb, a “C3 convertase”, that cleaves C3 into C3a and C3b. C3b also enters other complement pathways, promoting C5a release and cytolysis. FH destabilises C3b.Bb, competes with FB for binding to C3b, and is a cofactor for factor I (FI) that cleaves C3b to the CR3-ligand iC3b. Some FHRs likely antagonize FH.

Complement factor H (FH, encoded by *CFH*) is a soluble AP suppressor operating on autologous surfaces by recognising self-surfaces, directly *via* specific glycosaminoglycans and sialic acid or indirectly e.g. *via* C-reactive protein (CRP), and in fluid-phase. FH also aids non-inflammatory clearance of damaged cells and cell debris. Further “non-canonical” FH functions are mediated *via* binding sites, notably for complement receptor (CR3), malondialdehyde (MDA)-modified proteins and apolipoprotein E (apoE), distributed along its 20 CCP modules (CCPs) also called short consensus repeats (**Figure 1**). FH-like 1 (FHL-1) is an AP-regulating smaller splice variant that lacks self-*versus*-nonself discrimination. Six FH-related proteins (FHRs-1-3, 4A and 4B, and 5) complete the family (**Figure 1**). Encoded by a gene cluster situated *3’* of *CFH* (*CFHR3*;*CFHR1*;*CFHR4*;*CFHR2*;*CFHR5*), these products of genomic duplication events may antagonise FH.

In *CFH* and *CFHRs*, single-nucleotide polymorphisms, copy-number variations, and exon duplications, deletions and rearrangements are commonplace. These can alter risks of atypical haemolytic uraemic syndrome (aHUS), C3 glomerulopathy (C3G) or age-related macular degeneration (AMD) amongst many other diseases. In a fast-moving field, the 12 papers in this Research Topic take stock of efforts to understand how these proteins work, and sometimes fail.

The interplay between FH-family members is unpicked in Poppelaars et al. and Mannes et al. The C-terminal CCPs of FHRs resemble, and may compete with, the self-surface-recognising and C3b-binding CCPs 19-20 of FH. But CCPs equivalent to the C3b-binding CCPs 1-4 of FH, critical for AP regulation, are absent from FHRs. N-terminal CCPs in FHRs-1, 2 and 5 stabilise homo-/hetero-dimerisation (**Figure 1**). Poppelaars et al. discuss discrepancies in reported functions and serum-levels of FH-family proteins. Conversely, FHL-1 (reviewed by Mannes et al.) possesses the CCPs 1-4 of FH along with its CCPs 5-7 that augment their function, but lacks FH’s C-terminal CCPs, accounting for its lack of self/non-self discrimination. Being smaller, FHL-1 reaches locations poorly accessible to FH, *e.g.* by crossing Bruch’s membrane in the eye. These two reviews highlight challenges with working on FHRs and FHL-1 including shortages of specific antibodies and good animal models.

Proposed mechanisms for FH invoke simultaneous engagement of CCPs 1-4 and CCPs 19-20 of FH with the same C3b molecule, facilitated by 14 connecting CCPs (**Figure 1**). Dunne et al. identify a low-affinity dimerization site in CCPs 17-18, potentially explaining disease-linked mutations therein. Dimerisation could help FH molecules gather at sites requiring robust AP suppression. The role of FH’s sialylated N-glycans is incompletely understood. Delgado et al. describe desialylation of FH by bacterial neuraminidase in cases of *Streptococcus pneumoniae*-precipitated aHUS (*Sp*-aHUS). *In-vitro* enzymatic desialylation rendered FH less effective at preventing complement-mediated haemolysis, suggesting a pathological mechanism underlying *Sp*-aHUS. Rare genetic variations of the *CFH*/*CFHR* cluster, observed in many patients in this study, might also contribute to disease.

Screening for genetic variants, routine in aHUS management, is informed by knowledge of the consequences of specific mutations. Wong et al. describe a workflow for characterising “variants of unknown significance” identified in aHUS, C3G and AMD patients, within the AP-regulating CCPs 1-4 of FH. Of six new SNPs investigated, Q81P emerged as a dysfunctional, potentially causative, mutation in aHUS. Less impacted variants (*e.g.* D130N in this study) might contribute to AMD, a slower progressing disease. This brings to a useful total of 16 the number of biochemically characterised FH N-terminal variants.

Zhang et al. found FH autoantibodies (FH-AAs) in about 1-in-9 and 1-in-30 members of North American aHUS and C3G cohorts, respectively. Consistent with patterns of genetic variation, most FH-AAs in aHUS bind to and compromise CCPs 19-20, while C3G-associated FH-AAs often bind to and compromise N-terminal CCPs. Of aHUS patients with FH-AAs, ~75% were Δ*CFHR3*;*CFHR1* (*cf* ~3.5% of controls and in the C3G cohort). One hypothesis suggests C-terminal CCPs of FHR-1 (98% identity with FH CCPs 19-20) induce tolerance to a cryptic epitope in FH’s C-terminal region that becomes exposed during infection.

Autoantibodies binding CCPs 19-20 of FH and inhibiting C3b binding were also found in four of 45 cases of neuromyelitis optica spectrum disorder (Uzonyi et al.) a rare inflammatory disease of the CNS, also associated with other autoantibodies, that responds to therapeutic complement suppression. These FH-AAs cross-reacted with FHR-1, but none of these individuals is Δ*CFHR3*;*CFHR1*, or had kidney disease. Studies on larger cohorts will determine how the FH-AAs are generated and contribute to underlying pathophysiology.

By definition, successful human pathogens evade the AP. Hijacking by pathogens of FH for self-protection is achieved by many organisms; Moore et al. list >30 such bacterial species, plus fungi, protozoa, helminths and viruses. Pathogens utilise diverse molecules to anchor FH including, but not limited to, glycans and proteins that mimic host equivalents. FH-binding proteins of pathogens are often, although not invariably, important virulence factors with expression levels depending on the abundance of FH in the milieu. Antimicrobial strategies targeting FH capture include uses of sialic acid analogues, small molecules to lock-in non-FH binding conformations of FH-anchoring proteins, and – as illustrated by Laabei et al. - engineered FH-like molecules that displace host FH. In this case a chimeric protein, fusing the *Moraxella catarrhalis*-binding CCPs (6-7) of FH to IgG Fc, outcompeted FH for binding to its receptor on bacteria whereupon it activated classical complement *via* its Fc, promoting bacterial elimination.

Beyond its canonical roles in complement, surface-bound FH binds and influences the behaviour of neutrophils and monocytes. Research described in Kárpáti et al. extends this property to surface-immobilised FHL-1, FHR-1 and FHR-5, detailing how these proteins may affect extravasation and pathogen-killing by variously modulating adherence and migration, as well as production of IL-8, IL-1β, TNFα, and anti-inflammatory IL-10. A key challenge is to confirm the identity of the cell-surface FH/FHR receptor(s); CR3 is the lead candidate. In related work, Kozma et al. explored use of engineered FH to supress the cytokine storm potentially triggered by C3a/C5a binding to receptors on peripheral blood mononuclear cells. In cells cultured with autologous serum and subjected to artificial immune activation, a “mini”- FH, comprising CCPs 1-4 linked to CCPs 19-20, mildly suppressed IL-6 production and stimulated IL-10 production. Also of growing interest is how FH deters formation of lipid-rich deposits (LRD), and suppresses their inflammatory potential. As argued by Meri and Haapasalo, LRDs characterise not only AMD (drusen) and some forms of C3G (dense deposits) but also occur as plaques in Alzheimer’s disease and atherosclerosis. Noting that FH binds lipid-free and high-density lipoprotein-associated ApoE, oxidized lipids and bisretinoids in drusen, and malondialdehyde-conjugates (by-products of lipid peroxidation), these authors suggest potential involvement of aberrantly functioning FH in the pathophysiology underlying all these conditions.

Together, the articles in this Research Topic emphasise the pivotal importance of FH. The viability and health of our cells and tissues depend strongly on FH-mediated protection. The multitude of pathogenic microbes that exploit FH for their survival in human tissues attests to its role as the “master regulator” of the complement system. The growing list of diseases linked to variants or mutations in the *CFH* and *CFHR*s cluster underlines the need to develop better tools and animal models to aid in research, and supports establishment of units, within hospital laboratory settings, specialising in complement diagnostics.

## Author Contributions

All authors listed have made a substantial, direct, and intellectual contribution to the work and approved it for publication.

## Funding

MJ is supported by the National Research, Development and Innovation Office (OTKA grant K125219), the Hungarian Academy of Sciences (0106307), the Institutional Excellence Program to ELTE (NKFIH-1157/8/2019, D11206), the European Union's Horizon 2020 research and innovation programme under grant agreement No. 899163 (SciFiMed), the Kidneeds Foundation (Iowa, US), and the Ministry for Innovation and Technology from the Hungarian NRDI Fund (2020-1.1.6-JÖVŐ-2021-00010). PNB is supported by IBoIC 2020-3-3. SM is supported by the Sigrid Jusélius Foundation (4708373), State Funding to the Helsinki University Hospitals (VTR): TYH2019311 and HUS Diagnostic Center (Y780021099).

## Conflict of Interest

The authors declare that the research was conducted in the absence of any commercial or financial relationships that could be construed as a potential conflict of interest.

## Publisher’s Note

All claims expressed in this article are solely those of the authors and do not necessarily represent those of their affiliated organizations, or those of the publisher, the editors and the reviewers. Any product that may be evaluated in this article, or claim that may be made by its manufacturer, is not guaranteed or endorsed by the publisher.

